# Oral Frailty Is Associated With the Physical Component of Health‐Related Quality of Life in Middle‐Aged and Older Japanese Master Athletes

**DOI:** 10.1111/ggi.70889

**Published:** 2026-10-27

**Authors:** Eriko Nakata, Keizo Yamamoto, Shinobu Mitamura, Naoko Oda, Hiromi Niina, Aya Nohara, Yumiko Hibi, Aya Uemura, Kensuke Takezawa, Mami Fujibayashi

**Affiliations:** ^1^ Faculty of Agriculture Setsunan University Hirakata Osaka Japan; ^2^ Faculty of Contemporary Social Studies Setsunan University Neyagawa Osaka Japan; ^3^ Faculty of Pharmaceutical Sciences Setsunan University Hirakata Osaka Japan; ^4^ College of Gastronomy Management Ritsumeikan University Kusatsu Shiga Japan; ^5^ Hannan Chuo Hospital Matsubara Osaka Japan; ^6^ Division of Physical and Health Education Setsunan University Neyagawa Osaka Japan

**Keywords:** adults, health‐related quality of life, master athletes, oral frailty, oral health

## Abstract

**Background:**

The association between health‐related quality of life (HRQoL) and oral health remains unclear, especially in physically active adults. This study aimed to examine the association between HRQoL and oral frailty in middle‐aged and older Japanese master athletes.

**Methods:**

In this cross‐sectional study, 197 Japanese master athletes aged ≥ 35 years participated. HRQoL was assessed using the Short Form‐8, from which physical (PCS) and mental (MCS) component summary scores were derived. Oral frailty was assessed using the Oral Frailty 5‐Item Checklist. Multivariate regression analyses were conducted to adjust for potential confounders.

**Results:**

Of the participants, 195 individuals (median age: 59.0 years [interquartile range: 53.8–68.0], 41.0% women) were included in the analysis. The prevalence of oral frailty was 37.5% in PCS Tertile 1, compared with 16.9% in Tertile 2 and 16.7% in Tertile 3, while the prevalence was similar across MCS tertiles. Multivariate regression analysis revealed that oral frailty was independently and inversely associated with PCS (standardized *β* = −0.20, *p* = 0.006), but not MCS (standardized *β* = −0.12, *p* = 0.090). In the sub‐analysis, difficulty chewing (standardized *β* = −0.15, *p* = 0.034) and swallowing (standardized *β* = −0.26, *p* < 0.001) were associated with PCS, whereas fewer teeth (standardized *β* = −0.15, *p* = 0.035) and dry mouth (standardized *β* = −0.21, *p* = 0.004) were associated with MCS.

**Conclusion:**

Oral frailty was significantly associated with physical HRQoL in Japanese master athletes. These findings suggest that incorporating oral frailty assessment into health promotion strategies may enhance HRQoL in physically active adults.

## Graphical abstract

Among Japanese master athletes, oral frailty was independently associated with lower physical HRQoL, but not mental HRQoL. These findings suggest that oral frailty may be relevant to physical well‐being even in middle‐aged and older adults who maintain high levels of physical activity.

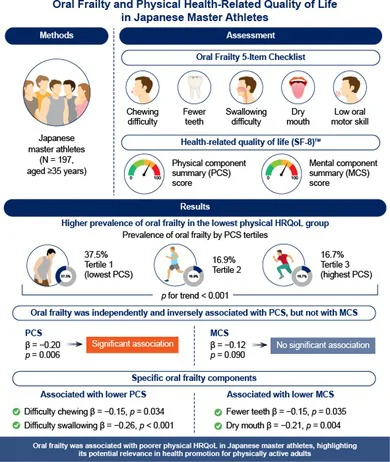

## 1 Introduction

As successful aging becomes increasingly important in society, physically active adults are attracting growing attention. In Japan, middle‐aged and older adults currently represent the highest proportion of those who exercise regularly, and this proportion has been steadily rising [1]. Supporting health of physically active adults may thus have a broader societal impact by exemplifying successful aging. Identifying their specific health challenges and developing targeted strategies is essential for public health.

Against this background, master athletes, defined as experienced athletes aged ≥ 35 years [2, 3], are often regarded as role models for healthy aging due to their superior physical performance [4]. However, they are not exempt from age‐related physiological changes and remain vulnerable to sports‐related injuries and cardiovascular conditions [3, 5].

Among these challenges, oral health decline is a growing concern that affects both the general and athletic populations [6]. Poor oral health, such as dental caries, periodontal disease, and tooth loss, not only affects oral conditions but also impacts systemic health, including non‐communicable and cardiovascular diseases [7, 8, 9]. Maintaining oral health is therefore a key component of overall health and successful aging in older adults.

“Oral frailty” is a concept introduced in Japan that offers a multidimensional evaluation of age‐related decline in oral function and has been proposed as an early indicator of functional decline in older adults [10]. It is characterized by subtle deterioration related to factors such as tooth loss, poor oral hygiene, inappropriate dental prosthetics, and difficulties in chewing or swallowing. Recognizing oral frailty aims to enable early intervention and prevent further decline in overall function [10]. Previous studies have reported that 14.3%–52.1% of middle‐aged and older Japanese exhibit signs of oral frailty [11, 12, 13].

As age‐related functional decline is closely reflected in quality of life, assessing the impact of oral frailty on health‐related quality of life (HRQoL) is particularly important in an aging society. While poor oral health, particularly oral frailty, has been associated with reduced HRQoL in the general population [14, 15], research in athletes remains limited and inconclusive [16, 17]. Furthermore, these studies [16, 17] have focused on single factors (e.g., missing teeth), overlooking the multidimensional aspects of oral health decline, including subjective symptoms such as dry mouth and chewing difficulty [18]. These approaches may not fully capture the association between oral health decline and quality of life, especially in populations that face complex age‐related changes, such as master athletes.

Moreover, most studies have predominantly focused on younger, elite athletes and did not consider the unique physiological and psychosocial characteristics of the aging athlete population. As a result, their findings may not be applicable to older master athletes.

Despite growing sports participation among middle‐aged and older adults, little is known about their oral health and its association with HRQoL. To address this gap, the present study aims to investigate the association between oral frailty and HRQoL among Japanese master athletes. Clarifying this association may provide scientific evidence for the development of oral health policies that support not only physical performance but also long‐term health in physically active aging populations. Furthermore, studying master athletes may have important international significance in geriatric medicine. Because they maintain high levels of physical activity into later life, this population provides a unique opportunity to distinguish age‐related changes from those attributable to physical inactivity. Although oral health behaviors and access to dental care vary across countries, identifying factors associated with well‐being in master athletes may provide broadly applicable insights for developing strategies to promote healthy and successful aging.

## 2 Methods

### 2.1 Design, Setting, and Participants

This cross‐sectional study determined the required sample size using G*Power 3.1.9.7 [19]. For the multivariate regression analysis, an effect size (*f* ^2^) of 0.15, an alpha error of 0.05, a power of 0.8, and approximately 10 explanatory variables were assumed, resulting in a required sample size of 119.

Participants were recruited on a voluntary basis through posters and flyers distributed at the 45th All‐Japan Masters Athletics Championships, held from September 21–23, 2024, in Kyoto, Japan.

The inclusion criteria were: (i) participation as an athlete in the competition and (ii) being 35 years of age or older [3]. The exclusion criteria were: (i) non‐athletes (e.g., family members or friends of competitors), (ii) individuals unable to complete the self‐administered questionnaire for physical or cognitive reasons, and (iii) those with missing data on HRQoL or oral frailty.

Athletes who were interested in participating visited a designated survey booth at the competition venue, where measurements and questionnaire data were collected.

This study was conducted in accordance with the Declaration of Helsinki and was approved by the research ethics committee of the authors' institution (approval number: 2024‐035, approval date: July 31, 2024). Before the survey, all participants were informed about the study's purpose and provided written informed consent.

### 2.2 Assessment of HRQoL

HRQoL was assessed using the Japanese version of the Medical Outcomes Study 8‐Item Short‐Form Health Survey (SF‐8) [20, 21], a validated instrument developed as a condensed version of SF‐36 [22]. The SF‐8 measures eight distinct health profile dimensions: physical function, role functioning‐physical, bodily pain, general health perception, vitality, social functioning, role functioning‐emotional, and mental health. The SF‐8 can calculate two summary scores, the physical component summary (PCS) and the mental component summary (MCS), both standardized to the Japanese national norm (mean = 50, SD = 10). Higher scores on these scales indicate better HRQoL. The license to use the SF‐8 instrument was obtained from Qualitest Co. Ltd., Japan.

### 2.3 Assessment of Oral Frailty Using the Oral Frailty 5‐Item Checklist

Oral frailty status was assessed using the validated Oral Frailty 5‐Item Checklist (OF‐5), which enables quick and easy evaluation of oral frailty without requiring specialized dental skills [23]. The OF‐5 comprises five components: (i) fewer teeth [23], (ii) difficulty chewing [24], (iii) difficulty swallowing [24], (iv) dry mouth [24], and (v) low articulatory oral motor skills [23]. Participants were classified as having oral frailty when declines were observed in two or more of these functions [23].

Fewer teeth was defined as having 0–19 natural teeth, based on the response to a structured question [23]. The remaining three subjective symptoms (difficulty chewing, difficulty swallowing, and dry mouth) were evaluated using items from the oral domain of Kihon checklist [24].

Articulatory oral motor skills were evaluated via oral diadochokinesis. Participants were instructed to pronounce the syllable “*Ta*” as clearly and rapidly as possible for 5 s. Trained researchers used the Kenko‐kun Handy (Takei Scientific Instruments Co. Ltd., Niigata, Japan) to count repetitions per second. Low articulatory oral motor skill was defined as fewer than 6.0 repetitions per second [23].

### 2.4 Other Variables

Height (cm) and weight (kg) were measured, and body mass index (BMI, kg/m^2^) was calculated. Grip strength (kgf) was measured on each hand alternately, and the average of the higher values for each hand was calculated.

A self‐administered questionnaire was used to collect data on sociodemographic characteristics (e.g., age, sex, marital status, education, and employment), lifestyle factors (drinking, smoking), medical history (diabetes, hypertension, and dyslipidemia), and athletic background (years of experience in track and field, a history of orthopedic visits).

Sleep duration in the past month was assessed using the Pittsburgh Sleep Quality Index [25]. Participants with a history of sports injuries requiring more than 1 week of rest in the past year were categorized accordingly [26]. Details of measurement instruments, variable categorization, and questionnaire items are provided in Supplementary Text A.

### 2.5 Statistical Analysis

Continuous variables are expressed as median [interquartile range, IQR], and qualitative variables are expressed as *n* (%). The Shapiro–Wilk test and histogram inspection confirmed non‐normal distributions. PCS and MCS were divided into tertiles for comparative analysis. Details of tertile cutoffs are provided in Supplementary Text B. The Jonckheere–Terpstra test and chi‐squared test were used to examine linear trends and proportions, respectively.

Multivariate regression analyses (forced entry method) were performed to evaluate associations between oral frailty and PCS/MCS in the complete‐case analysis. Potential confounding factors for the association between oral frailty and QoL, as well as factors previously reported to be associated with PCS or MCS, were included as covariates in the respective multivariable regression models as follows: PCS: age, sex, BMI, employment status [27], a history of orthopedic visits [28], years of experience in track and field [29]; MCS: age, sex, BMI, marital status, diabetes, sleep duration, and a history of injury‐related sports interruptions [30]. To examine in detail which of the five components of oral frailty are particularly related to HRQoL, the sub‐analysis examined the association of each of the five oral frailty components with PCS and MCS.

Multicollinearity was assessed using the variance inflation factor (VIF), with VIF ≥ 10 indicating multicollinearity. Model fit was evaluated using the coefficient of determination (*R* ^2^).

Additionally, missing values were observed in the covariates. The number of missing values for each variable is presented in Supplementary Table 1. To assess the robustness of the findings to the handling of missing data, multiple imputation was performed as a sensitivity analysis under the missing‐at‐random assumption. Five datasets (*m* = 5) were generated using the method of multivariate imputation by chained equations (MICE). Multivariate linear regression analysis was conducted on each imputed dataset. Regression coefficients, 95% confidence intervals, and *p*‐values were pooled across imputed datasets using Rubin's rules. Standardized regression coefficients were not reported because pooled standardized coefficients were not provided by the standard multiple imputation procedure in SPSS. Therefore, pooled regression coefficients and 95% confidence intervals were used to assess the robustness of the findings.

Data were analyzed using SPSS Statistics version 27 for the primary analysis and SPSS Missing Value version 32 for multivariate imputation (IBM Corp., Armonk, NY, USA). All tests were two‐tailed with a test level of *α* = 0.05.

### 2.6 AI Tools Disclosure

This manuscript was prepared with the assistance of DeepL and ChatGPT to improve linguistic readability.

## 3 Results

### 3.1 Participants' Characteristics

A total of 197 athletes participated, and after excluding two with missing data on HRQoL, 195 were included in the analysis (median age: 59.0 years [IQR: 53.8–68.0]; 41.0% women, Figure 1). The median PCS and MCS scores were 49.8 [46.0–53.1] and 53.6 [49.7–55.8], respectively. Oral frailty was identified in 46 participants (23.6%).

**FIGURE 1:**
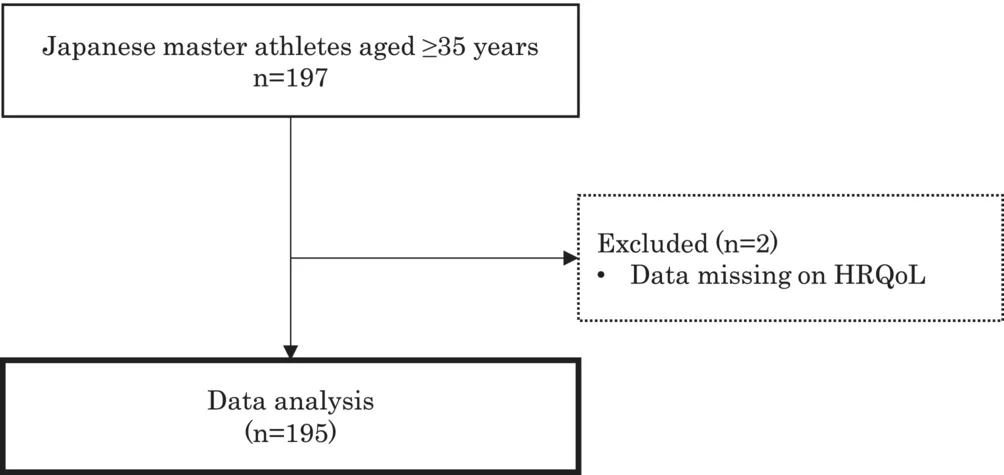
Participant flow diagram.

Table 1 shows the comparison of oral frailty, its components, and participant characteristics across the three PCS and MCS tertiles. The prevalence of oral frailty was higher in PCS Tertile 1 (37.5%) than in Tertiles 2 (16.9%) and 3 (16.7%). The prevalence of difficulty swallowing was also higher in PCS Tertile 1 (28.1%) than in Tertiles 2 (6.2%) and 3 (4.5%), as was the prevalence of dry mouth (31.3%, 15.4%, and 16.7%, respectively). The prevalence of oral frailty was similar across MCS Tertiles 1, 2, and 3 (30.2%, 20.0%, and 20.9%, respectively), whereas dry mouth was more prevalent in MCS Tertile 1 (36.5%) than in Tertiles 2 (16.9%) and 3 (10.4%).

**TABLE 1:** Comparison of oral frailty status and other characteristics across PCS and MCS tertiles of HRQoL.

	Valid response	All	PCS				MCS			
			Tertile 1 (< 47.6) *n* = 64	Tertile 2 (47.6–51.6) *n* = 65	Tertile 3 (51.6 <) *n* = 66	*p* ^a^	Tertile 1 (< 50.6) *n* = 63	Tertile 2 (50.6–54.8) *n* = 65	Tertile 3 (54.8 <) *n* = 67	*p* ^a^
Oral frailty (presence), *n* (%)	195	46 (23.6)	24 (37.5)	11 (16.9)	11 (16.7)	0.006	19 (30.2)	13 (20)	14 (20.9)	0.326
Fewer teeth, *n* (%)	195	52 (26.7)	16 (25.0)	15 (23.1)	21 (31.8)	0.493	21 (33.3)	13 (20.0)	18 (26.9)	0.233
Difficulty chewing (yes), *n* (%)	195	27 (13.8)	12 (18.8)	7 (10.8)	8 (12.1)	0.373	10 (15.9)	9 (13.8)	8 (11.9)	0.810
Difficulty swallowing (yes), *n* (%)	195	25 (12.8)	18 (28.1)	4 (6.2)	3 (4.5)	< 0.001	11 (17.5)	7 (10.8)	7 (10.4)	0.407
Dry mouth (yes), *n* (%)	195	41 (21.0)	20 (31.3)	10 (15.4)	11 (16.7)	0.049	23 (36.5)	11 (16.9)	7 (10.4)	0.001
Oral diadochokinesis, times/s	195	7.0 (6.4:7.6)	7.1 (6.6:7.8)	7.0 (6.4:7.6)	7.0 (6.4:7.6)	0.640	7.2 (6.5:7.9)	7.0 (6.6:7.4)	7.0 (6.2:7.6)	0.118
Low articulatory oral motor skills (Oral diadochokinesis < 6.0 times/s), *n* (%)	195	23 (11.8)	10 (15.6)	8 (12.3)	5 (7.6)	0.359	6 (9.5)	5 (7.7)	12 (17.9)	0.152
Age, years	195	59.0 (53.8:68.0)	57.0 (52:67.3)	59.0 (54.5:69.0)	61.0 (54.0:68.0)	0.426	55.0 (51.5:64.0)	61.0 (55.0:70.0)	62.0 (55.0:71.0)	0.002
Sex: women, *n* (%)	195	80 (41.0)	28 (43.8)	25 (38.5)	27 (40.9)	0.191	31 (49.2)	29 (44.6)	20 (29.9)	0.062
Body mass index, kg/m^2^	194	21.3 (19.8:22.7)	21.2 (19.8:22.3)	21.6 (20.0:23.0)	20.8 (19.4:22.7)	0.803	21.2 (19.8:22.6)	21.3 (19.2:22.8)	21.7 (20.0:22.7)	0.164
Grip strength, kgf	194	34.4 (27:41.6)	35.9 (26.1:41.4)	34.2 (27.1:43.6)	31.7 (27.0:40.9)	0.530	34.1 (27.2:41.4)	31.5 (26.3:44.7)	36.5 (27.0:45.2)	0.161
Drinking (≥ 3 times/week), *n* (%)	195	65 (33.3)	23 (35.9)	22 (33.8)	20 (30.3)	0.788	21 (33.3)	17 (26.2)	27 (40.3)	0.226
Smoking (current/former), *n* (%)	195	19 (9.7)	4 (6.3)	5 (7.7)	10 (15.2)	0.183	7 (11.1)	4 (6.2)	8 (11.9)	0.483
Marital status (others), *n* (%)^b^	194	28 (14.4)	11 (17.2)	9 (13.8)	8 (12.1)	0.680	14 (22.2)	9 (13.8)	5 (7.5)	0.060
Employment status (not employed), *n* (%)	194	40 (20.5)	8 (12.5)	18 (27.7)	14 (21.2)	0.110	9 (14.3)	15 (23.1)	16 (23.9)	0.315
Educational background (“junior high school graduate or below”), *n* (%)	181	3 (1.5)	1 (1.6)	1 (1.5)	1 (1.5)	1.000	0 0	2 (3.1)	1 (1.5)	0.381
Medical history, *n* (%)	195									
Diabetes	195	16 (8.2)	3 (4.7)	8 (12.3)	5 (7.6)	0.281	8 (12.7)	6 (9.2)	2 (3.0)	0.122
Hypertension	195	20 (10.3)	8 (12.5)	8 (12.3)	4 (6.1)	0.385	7 (11.1)	5 (7.7)	8 (11.9)	0.697
Dyslipidemia	195	13 (6.7)	6 (9.4)	4 (6.2)	3 (4.5)	0.533	4 (6.3)	4 (6.2)	5 (7.5)	0.948
Years of experience in track and field, years	188	20.0 (9.0:39.0)	26.0 (8.5:41.0)	24.0 (10.5:40.0)	14.0 (6.0:34.0)	0.038	15.0 (5.5:35.0)	20.0 (10.0:40.8)	24.0 (9.0:39.0)	0.412
Sleep duration, hours	195	6.0 (6.0:7.0)	6.0 (6.0:7.0)	6.5 (5.8:7.0)	6.5 (5.5:7.0)	0.477	6.0 (5.3:6.8)	6.9 (6.0:7.0)	6.5 (6.0:7.0)	0.015
A history of orthopedic visits, *n* (%)	195	13 (6.7)	8 (12.5)	2 (3.1)	3 (4.5)	0.070	4 (6.3)	6 (9.2)	3 (4.5)	0.545
A history of injury‐related sports interruptions, *n* (%)^c^	194	80 (41.0)	20 (31.3)	29 (44.6)	31 (47.0)	0.170	24 (38.1)	27 (41.5)	29 (43.3)	0.820

*Note:* Data described the median (25 percentile: 75 percentile) for continuous variables and *n* (%) for qualitative variables.

Abbreviations: HRQoL, health‐related quality of life; MCS, mental component summary; PCS, physical component summary.

^a^ The Jonckheere‐Terpstra test was used to confirm linear trends in continuous variables and the chi‐square test was used to compare rates of qualitative variables.

^b^ Others: Bereaved/divorced/never married.

^c^ Interruption of > 1 week due to injury in past year.

Participants in PCS Tertile 1 had more years of experience in track and field (26.0 years) than those in Tertiles 2 (24.0 years) and 3 (14.0 years). A history of orthopedic visits was more common in PCS Tertile 1 (12.5%) than in Tertiles 2 (3.1%) and 3 (4.5%).

Participants in MCS Tertile 1 were younger (55.0 years) than those in Tertiles 2 (61.0 years) and 3 (62.0 years) and had a shorter sleep duration (6.0, 6.9, and 6.5 h, respectively). Additionally, the proportions of female participants (49.2%, 44.6%, and 29.9%, respectively) and unmarried participants (22.2%, 13.8%, and 7.5%, respectively) were also higher in MCS Tertile 1 than in Tertiles 2 and 3.

### 3.2 Multivariate Regression Analysis: PCS

Table 2 shows the multivariate regression analysis results for PCS. In the model after adjusting for confounders, oral frailty showed an independent association to lower PCS (standardized *β* = −0.20, *p* = 0.006). The model explained 13.7% of the variance (*p* for ANOVA < 0.001).

**TABLE 2:** Multivariate regression analysis (forced entry method) for the association between physical component summary, oral frailty, and covariates (*n* = 187).

	*B*	Standardized *β*	95% confidence interval		*p* ^a^
			Lower limit	Upper limit	
Oral frailty (presence = 1, absence = 0)	−2.69	−0.20	−4.58	−0.80	0.006
Age (per 1.0 year)	0.03	0.05	−0.06	0.11	0.549
Sex (women = 1, men = 0)	0.00	0.00	−1.75	1.76	0.997
Body mass index (per 0.1 kg/m^2^)	0.15	0.08	−0.12	0.42	0.285
Employment status (not employed = 1, employed = 0)	−1.31	−0.09	−3.68	1.06	0.276
Years of experience in track and field (per 1 year)	−0.03	−0.06	−0.07	0.02	0.197
A history of orthopedic visits (presence = 1, absence = 0)	−7.11	−0.31	−10.18	−4.04	< 0.001
*R* ^2^	0.137				
*F*	5.215				
*p* ^b^	< 0.001				

^a^ *p* for independent variables.

^b^ *p* for analysis of variance.

### 3.3 Multivariate Regression Analysis: MCS

Table 3 shows the multivariate regression analysis results for MCS. In the model after adjusting for confounders, oral frailty was not significantly associated with MCS (standardized *β* = −0.12, *p* = 0.090). The model explained 13.5% of the variance (*p* for ANOVA < 0.001).

**TABLE 3:** Multivariate regression analysis (forced entry method) for the association between mental component summary, oral frailty, and covariates (*n* = 193).

	*B*	Standardized *β*	95% confidence interval		*p* ^a^
			Lower limit	Upper limit	
Oral frailty (presence = 1, absence = 0)	−1.51	−0.12	−3.26	0.24	0.090
Age (per 1.0 year)	0.10	0.21	0.03	0.16	0.005
Sex (women = 1, men = 0)	−1.37	−0.13	−2.96	0.22	0.092
Body mass index (per 0.1 kg/m^2^)	0.05	0.03	−0.20	0.30	0.707
Marital status (others^c^ = 1, married = 0)	−3.23	−0.22	−5.26	−1.19	0.002
Diabetes (presence = 1, absence = 0)	−2.98	−0.16	−5.73	−0.24	0.033
A history of injury‐related sports interruptions (presence = 1, absence = 0)^d^	1.02	0.10	−0.46	2.50	0.176
Sleep duration (per 1.0 h)	0.42	0.09	−0.25	1.10	0.217
*R* ^2^	0.135				
*F*	4.740				
*p* ^b^	< 0.001				

^a^ *p* for independent variables.

^b^ *p* for analysis of variance.

^c^ Bereaved/divorced/never married.

^d^ Interruption of > 1 week due to injury in past year.

### 3.4 Sub‐Analysis: Oral Frailty Components and HRQoL

Table 4 shows the results of the sub‐analysis examining each component of oral frailty. For PCS, difficulty chewing (standardized *β* = −0.15, *p* = 0.034) and difficulty swallowing (standardized *β* = −0.26, *p* < 0.001) were negatively associated. For MCS, fewer teeth (standardized *β* = −0.15, *p* = 0.035) and dry mouth (standardized *β* = −0.21, *p* = 0.004) were both negatively associated.

**TABLE 4:** Multivariate regression analyses for the association between HRQoL and oral frailty components (PCS: *n* = 187, MCS: *n* = 193).

	PCS^a^					*R* ^2^	*F*	*p* ^d^	MCS^b^					*R* ^2^	*F*	*p* ^d^
	*B*	Standardized *β*	95% CI		*p* ^c^				*B*	Standardized *β*	95% CI		*p* ^c^			
			Lower limit	Upper limit							Lower limit	Upper limit				
Fewer teeth (< 20 = 1, ≥ 20 = 0)	0.73	0.06	−1.09	2.55	0.429	0.102	4.022	< 0.001	−1.76	−0.15	−3.4	−0.13	0.035	0.142	4.979	< 0.001
Difficulty chewing (yes = 1, no = 0)	−2.57	−0.15	−4.94	−0.2	0.034	0.121	4.671	< 0.001	−0.74	−0.05	−2.9	1.43	0.502	0.123	4.377	< 0.001
Difficulty swallowing (yes = 1, no = 0)	−4.57	−0.26	−6.93	−2.21	< 0.001	0.167	6.324	< 0.001	−1.77	−0.11	−3.87	0.34	0.100	0.134	4.716	< 0.001
Dry mouth (yes = 1, no = 0)	−1.69	−0.12	−3.7	0.33	0.101	0.112	4.368	< 0.001	−2.77	−0.21	−4.63	−0.91	0.004	0.16	5.586	< 0.001
Low articulatory oral motor skills (presence = 1, absence = 0)	−1.43	−0.08	−4.03	1.17	0.279	0.105	4.113	< 0.001	−0.38	−0.02	−2.71	1.94	0.745	0.122	4.326	< 0.001

Abbreviations: CI, confidence interval; HRQoL, health‐related quality of life; MCS, mental component summary; PCS, physical component summary.

^a^ Covariates included age, sex, body mass index, employment status, a history of orthopedic visits, and years of experience in track and field.

^b^ Covariates included age, sex, body mass index, marital status, diabetes, a history of injury‐related sports interruptions, and sleep duration.

^c^ *p* for independent variables.

^d^ *p* for analysis of variance.

### 3.5 Sensitivity Analysis

In the sensitivity analysis using multiple imputation, the association between oral frailty and its components and HRQoL were materially unchanged from that observed in the complete‐case analysis. Oral frailty was negatively associated with PCS (*B* = −2.88, 95% confidence interval: −4.75 to −1.00, *p* = 0.003), whereas no statistically significant association was observed between oral frailty and MCS (*B* = −1.57, 95% confidence interval: −3.29 to 0.15, *p* = 0.073). Detailed results are available in Supplementary Tables 2–4.

## 4 Discussion

To our knowledge, this is the first study to examine the association between oral frailty and HRQoL in master athletes, a population that has received little attention to date. Oral frailty was identified in 23.6% of participants and was significantly associated with lower PCS, even after adjusting for confounders. Sub‐analysis showed that chewing and swallowing difficulties were associated with lower PCS, while fewer teeth and dry mouth were associated with lower MCS.

Regarding PCS, while findings in younger athletes have been inconsistent [16, 17], our findings were consistent with previous studies in non‐athletic adults showing that poor oral health, such as oral frailty, tooth loss, and dry mouth, is associated with reduced HRQoL [14, 15]. Furthermore, sensitivity analysis using the multiple imputation method also demonstrated effect sizes and directions similar to those of the original dataset, thereby confirming the robustness of our results. This suggests that not only oral frailty may be an important factor in aging even among master athletes, who are generally perceived as healthy, but also that high levels of physical activity may not be sufficient to offset the negative association between oral frailty and general health. Therefore, the prevention of oral frailty and the promotion of oral health should be an essential element of the care of physically active adults.

A possible mechanism is that, as with non‐athletes, oral frailty has been linked to muscle weakness [31] and physical function decline [32], which may contribute to lower PCS. Furthermore, considering the characteristics of master athletes, factors specific to athletes to consider include regular consumption of sports drinks [33], reduced saliva secretion during exercise [34], exercise‐induced immunodepression [35], varying levels of knowledge and prioritization regarding oral hygiene [6]. These factors, together with age‐related changes, may contribute to the development of oral frailty, which in turn may be associated with poorer nutritional status and overall health, potentially explaining its association with lower PCS. The mechanisms underlying the association between oral frailty and PCS in master athletes need to be elucidated in future studies.

On the other hand, despite reports that oral function is closely associated with mental health [36, 37, 38], no association was found with MCS. One reason for this may be that the association between oral function and mental health may be diluted by using oral frailty, an indicator that comprehensively assesses multiple aspects of oral function decline. In fact, our sub‐analysis showed a significant association between fewer teeth, dry mouth, and MCS. This suggests that decline in mental HRQoL may be related to specific oral functions, such as fewer teeth and dry mouth, rather than to the accumulation of oral function decline such as oral frailty. Our findings suggest that, while oral frailty is associated with poor physical health among master athletes, certain oral functions may also serve as indicators of poor mental health.

Notably, subjective oral functions, such as difficulty chewing, difficulty swallowing, and dry mouth which showed associations with HRQoL in the sub‐analysis, are the factors that can be modified through appropriate management and oral training [39]. Therefore, HRQoL of master athletes may be enhanced through interventions targeting oral frailty. In contrast, objective articulatory oral motor skills were not associated with HRQoL, unlike subjective oral functions. One possible explanation is that master athletes may maintain relatively good muscle strength in the stomatognathic system through daily physical activity and exercise [40, 41]. Although a direct comparison is not possible in this study, the median articulatory oral motor skill score in this study was higher than that reported in the general population (6.2) [42]. This preserved oral motor skills may have masked any associations with HRQoL. These findings suggest that among physically active adults, subjective oral function may be an important factor in assessing HRQoL.

A strength of this study is that it focused on master athletes, a population that has received little attention in the field of oral health, and demonstrated that oral frailty was associated with physical HRQoL even among individuals who maintain high levels of physical activity. This finding suggests that the association between oral frailty and HRQoL may not be limited to older adults with low levels of physical activity. Thus, our findings highlight the importance of assessing oral frailty and maintaining good oral health to support successful aging, even in physically active populations. Further longitudinal studies are needed to confirm these associations and inform evidence‐based interventions.

This study has several limitations. First, its cross‐sectional design precludes causal inference. Second, as the sample was limited to participants in a single competition, the generalizability of the findings may be restricted. Third, considering that the participants were a relatively healthy population, selection bias cannot be ruled out. Therefore, the findings should be interpreted with caution. Fourth, important potential confounding factors, such as dental and periodontal health, oral hygiene, socioeconomic status, and mental health, were not assessed. Fifth, the number of remaining teeth was self‐reported, although prior research suggests acceptable validity [43]. Finally, the distribution of oral frailty among master athletes could not be directly compared with that of non‐athletes because this study did not include a non‐athlete comparison group. Although a lower prevalence was observed than that reported among non‐athletic Japanese older adults [23], further studies are needed to clarify the characteristics of oral frailty in master athletes through direct comparison with appropriate control groups.

## 5 Conclusion

In Japanese master athletes, oral frailty was significantly associated with physical HRQoL. In addition, specific subcomponents of oral frailty were associated with mental HRQoL. These findings suggest that incorporating oral frailty assessment into health promotion strategies may enhance HRQoL in physically active adults. Further longitudinal studies are needed to confirm these associations and inform evidence‐based interventions.

## Author Contributions

Conceptualization: E.N. and M.F. Data curation: E.N., S.M., N.O., H.N., A.N., Y.H., A.U., and M.F. Formal analysis: E.N. and K.Y. Funding acquisition: E.N. Investigation: E.N., S.M., N.O., H.N., A.N., Y.H., A.U., and M.F. Methodology: E.N., S.M., N.O., H.N., A.N., Y.H., K.T., and M.F. Project administration: M.F. Resources: E.N., S.M., N.O., H.N., A.N., Y.H., and M.F. Supervision: M.F. Visualization: E.N. Writing – original draft: E.N., K.Y., and M.F. Writing – review and editing: E.N., K.Y., and M.F.

## Funding

This research was supported by KAKENHI [24K13252], a Grant‐in‐Aid for Scientific Research C, funded by the Japan Society for the Promotion of Science of Japan. The funding source had no role in the study design; in the collection, analysis, or interpretation of data; in the writing of the manuscript; or in the decision to submit the manuscript for publication.

## Ethics Statement

The study was approved by Setsunan University Ethical Review Committee for Medical and Health Research Involving Human Subjects (approval number: 2024‐035, approval date: July 31, 2024) and informed consent was obtained.

## Consent

The authors have nothing to report.

## Conflicts of Interest

The authors declare no conflicts of interest.

## Supporting information


**Supplementary Table 1:** The number of missing values for each variable.
**Supplementary Table 2:** Multivariate regression analysis (forced entry method) for the association between PCS, oral frailty, and covariates using multiple imputation.
**Supplementary Table 3:** Multivariate regression analysis (forced entry method) for the association between MCS, oral frailty, and covariates using multiple imputation.
**Supplementary Table 4:** Multivariate regression analyses for the association between HRQoL and oral frailty components using multiple imputation.

## Data Availability

The data that support the findings of this study are available from the corresponding author upon reasonable request.
