# Supplementary material for: Oral Frailty Is Associated With the Physical Component of Health‐Related Quality of Life in Middle‐Aged and Older Japanese Master Athletes
Source: Geriatr Gerontol Int. 2026 Oct 27;26(11):e70889. doi: 10.1111/ggi.70889 (PMC13714333; doi:10.1111/ggi.70889)
Supplement: Supplementary file 1 [file GGI-26-0-s001.docx]

**Supplementary Text A.**　 Details of Other Measurements and Variables

**Height and weight**: Measured using seca 213l (Seca Co., Ltd., cm) and HD-760-WH (TANITA Co., Ltd., kg), respectively.

**Grip strength**: Measured using TKK5401(Takei Kiki Co., Ltd.).

**Sociodemographic/lifestyle**:

- **Marital status**: married vs. others (bereaved/divorced/never married)
- **Education**: high school graduate or above vs. junior high school graduate or below
- **Employment**: employed vs. not employed
- **Drinking**: ≥3 times/week vs. <3 times/week
- **Smoking**: current/former vs. never

**Medical history**: Diabetes, hypertension, dyslipidemia (self-report)

**Sleep duration**: Participants responded to the question about their sleep duration over the past month using the Pittsburgh Sleep Quality Index.^2^

**A history of injury-related sports interruptions**: Interruption of >1 week due to injury in past year ^3^

**Supplementary Text B.**　 Tertile Cutoffs

PCS: tertile 1 (<47.6, n=64), tertile 2 (47.6–51.6, n=65), and tertile 3 (>51.6, n=65)

MCS: tertile 1 (<50.6, n=63), tertile 2 (50.6–54.8, n=65), and tertile 3 (54.8<, n=67)

| **Supplementary Table 1 The number of missing values for each variable** | | |
| --- | --- | --- |
|  | Missing values | |
|  | n | % |
| Oral frailty | 0 | 0.0 |
| Fewer teeth | 0 | 0.0 |
| Difficulty chewing | 0 | 0.0 |
| Difficulty swallowing | 0 | 0.0 |
| Dry mouth | 0 | 0.0 |
| Oral diadochokinesis | 0 | 0.0 |
| Low articulatory oral motor skills | 0 | 0.0 |
| Age | 0 | 0.0 |
| Sex: women | 0 | 0.0 |
| Body mass index | 1 | 0.5 |
| Grip strength | 1 | 0.5 |
| Drinking | 0 | 0.0 |
| Smoking | 0 | 0.0 |
| Marital status | 1 | 0.5 |
| Employment status | 1 | 0.5 |
| Educational background | 14 | 7.2 |
| Medical history |  |  |
| Diabetes | 0 | 0.0 |
| Hypertension | 0 | 0.0 |
| Dyslipidemia | 0 | 0.0 |
| Years of experience in track and field | 7 | 3.6 |
| Sleep duration | 0 | 0.0 |
| A history of orthopedic visits | 0 | 0.0 |
| A history of injury-related sports interruptions | 1 | 0.5 |

| **Supplementary Table 2 Multivariate regression analysis (forced entry method) for the association between PCS, oral frailty, and covariates using multiple imputation.** | | | | | |
| --- | --- | --- | --- | --- | --- |
|  |  | B | 95% confidence interval | | *p* |
|  |  |  | Lower Limit | Upper Limit |  |
| Oral frailty (presence=1, absence=0) | | -2.88 | -4.75 | -1.00 | 0.003 |
| Age (per 1.0 year) | | 0.03 | -0.05 | 0.12 | 0.450 |
| Sex (women=1, men=0) | | -0.19 | -1.9 | 1.53 | 0.832 |
| Body mass index (per 0.1 kg/m^2^) | | 0.07 | -0.19 | 0.33 | 0.617 |
| Employment status (Not employed=1, employed=0) | | 1.12 | -1.18 | 3.42 | 0.339 |
| Years of experience in track and field (per 1 year) | | -0.04 | -0.08 | 0.01 | 0.115 |
| A history of orthopedic visits (presence=1, absence=0) | | -6.97 | -10.04 | -3.91 | <0.001 |

PCS: physical component summary.

| **Supplementary Table 3 Multivariate regression analysis (forced entry method) for the association between MCS, oral frailty, and covariates using multiple imputation.** | | | | | |
| --- | --- | --- | --- | --- | --- |
|  |  | B | 95% confidence interval | | *p* |
|  |  |  | Lower Limit | Upper Limit |  |
| Oral frailty (presence=1, absence=0) | | -1.57 | -3.29 | 0.15 | 0.073 |
| Age (per 1.0 year) | | 0.09 | 0.03 | 0.16 | 0.005 |
| Sex (women=1, men=0) | | -1.41 | -2.98 | 0.16 | 0.078 |
| Body mass index (per 0.1 kg/m^2^) | | 0.04 | -0.2 | 0.29 | 0.727 |
| Marital status (others ^a^ =1, married=0) | | -3.25 | -5.26 | -1.23 | 0.002 |
| Diabetes (presence=1, absence=0) | | -2.98 | -5.69 | -0.26 | 0.032 |
| A history of injury-related sports interruptions (presence=1, absence=0) ^b^ | | 0.97 | -0.50 | 2.43 | 0.196 |
| Sleep duration (per 1.0 hour) | | 0.42 | -0.25 | 1.09 | 0.215 |
| ^a^ | bereaved/divorced/never married | | | | |
| ^b^ | interruption of >1 week due to injury in past year | | | | |

MCS: mental component summary.

| **Supplementary Table 4 Multivariate regression analyses for the association between HRQoL and oral frailty components using multiple imputation.** | | | | | | | | | | |
| --- | --- | --- | --- | --- | --- | --- | --- | --- | --- | --- |
|  |  | PCS^a^ | | | |  | MCS^b^ | | | |
|  |  | B | 95%CI | | *p* |  | B | 95%CI | | *p* |
|  |  |  | Lower | Upper |  |  |  | Lower | Upper |  |
|  |  |  | Limit | Limit |  |  |  | Limit | Limit |  |
| Fewer teeth (<20=1, ≥20=0) | | 0.55 | -1.26 | 2.35 | 0.553 |  | -1.78 | -3.40 | -0.16 | 0.031 |
| Difficulty chewing (yes=1, no=0) | | -2.93 | -5.27 | -0.59 | 0.014 |  | -0.82 | -2.93 | 1.29 | 0.445 |
| Difficulty swallowing (yes=1, no=0) | | -4.90 | -7.15 | -2.65 | <0.001 |  | -1.79 | -3.88 | 0.30 | 0.094 |
| Dry mouth (yes=1, no=0) | | -1.88 | -3.87 | 0.10 | 0.063 |  | -2.78 | -4.60 | -0.96 | 0.003 |
| Low articulatory oral motor skills (presence=1, absence=0) | | -1.03 | -3.58 | 1.52 | 0.429 |  | -0.41 | -2.71 | 1.90 | 0.729 |
| ^a^ | Covariates included age, sex, body mass index, employment status, a history of orthopedic visits, and years of experience in track and field. | | | | | | | | | |
| ^b^ | Covariates included age, sex, body mass index, marital status, diabetes, a history of injury-related sports interruptions, and sleep duration. | | | | | | | | | |
| HRQoL: health-related quality of life PCS: physical component summary MCS: mental component summary CI: confidence interval SE: Standard Errors | | | | | | | | | | |

**References**

1. G.A. Tayrose, B.G. Beutel, D.A. Cardone, and O.H. Sherman, “The masters athlete: a review of current exercise and treatment recommendations,” *Sports Health* 7, no. 3 (2015): 270–276, https://doi.org/10.1177/1941738114548999.

2. J.S. Carpenter and M.A. Andrykowski, “Psychometric evaluation of the Pittsburgh Sleep Quality Index,” *Journal of Psychosomatic Research* 45, no. 1 (1998): 5–13, https://doi.org/10.1016/S0022-3999(97)00298-5.

3. J. Kishi, “Relationship between athletic injury and mental health,” *Journal of Gifu Kyoritsu University* 46 (2013): 56–62. (in Japanese)
